# Thioacetamide-Induced Acute Hepatic Encephalopathy in Rat: Behavioral, Biochemical and Histological Changes

**Published:** 2012-03-01

**Authors:** M Farjam, P Dehdab, F Abbassnia, D Mehrabani, N Tanideh, S Pakbaz, M H Imanieh

**Affiliations:** 1 Department of Pharmacology, Shiraz University of Medical Sciences, Shiraz, Iran; 2 Fars Science Research Branch, Islamic Azad University, Shiraz, Iran; 3 Stem Cell Research and Transgenic Technology Research Center, Shiraz University of Medical Sciences, Shiraz, Iran; 4 Department of Pathology, Shiraz University of Medical Sciences, Shiraz, Iran; 5 Department of Pediatrics, Gastroenterohepatology Research Center, Shiraz University of Medical Sciences, Shiraz, Iran

**Keywords:** Acute hepatic encephalopathy, Thioacetamide, Rat

## Abstract

**Background:**

As a serious neuropsychiatric disease, hepatic encephalopathy (HE) is a clinical condition with several types regarding chronicity and clinical diversity that can develop as a complication of both acute and chronic liver failure. This study evaluates changes in thioacetamide (TAA)-induced acute hepatic encephalopathy (AHE) in rat as an animal model.

**Methods:**

Both genders of C57BL6, BALB/C mice and Sprague Dawley rats; (10 animals in each group) were compared for induction of AHE to clarify which animal and gender were appropriate. The animals (10 male rats in each group) were categorized in 4 groups according to the dose of the TAA administered (200, 300 and 400 mg/kg of TAA at 24 h intervals for 4 days). A control group was treated with solvent of TAA which was water (5 ml/kg/day). The behavioral, biochemical markers of hepatic failure and histological aspects of thioacetamide (TAA) induced AHE and the correlation between the clinical severity and liver failure biomarkers were evaluated.

**Results:**

Rat was shown to be an animal model of choice for AHE while the optimum dosage of TAA to induce AHE was 300 mg/kg/day at 24 h intervals for 4 days. The behavioral score was partially correlated with the rising of some biomarkers and pathological findings.

**Conclusion:**

Rat can be introduced as the animal of choice for AHE to study the pathophysiology, pharmacology and the survival rate of disease in liver transplant patients.

## Introduction

Hepatic encephalopathy (HE) is a clinical condition with several types regarding chronicity and clinical diversity.1 This syndrome can develop as a complication of both acute and chronic liver failure.[1][2] The wide spectrum of the clinical presentations add to the complexity of HE.[3][4] Meanwhile the burden of the disease on patients, family and health organization is still high.[5]

To include both types of hepatic abnormality and the characteristics of the neurological manifestations, a multiaxial definition has been accepted.[2] According to this definition, type A represents HE in patients with acute liver failure (ALF), type B is rare and was defined to be the neuropsychiatric complication of portal-systemic bypass without any intrinsic hepatocellular pathology and type C is the involvement of the brain seen in cirrhotic patients.[6]

Encephalopathy is a hallmark symptom in patients suffering from acute liver insufficiency and may progress from altered mental status to coma within days.[2] There is a very high rate of mortality in this type.[7][8][9] Supportive care until spontaneous recovery is the only treatment strategy but does not occur in many patients.[9][10] To prevent death, liver transplantation is the only effective approach. In practice, however; due to little time to prepare the patient and liver for transplantation, the death can occur.[6] This could be a problem even for a well-known transplant center as the center in Shiraz Nemazee Hospital.[11][12]

In contrast to type A, type C does not cause the patient’s demise although it carries a poor prognosis.[3][13] Therefore, a novel treatment for acute hepatic encephalopathy seems necessary to increase the survival of patients and improve the prognosis. Studies of the pathogenesis of human disorders have been significantly improved by utilization of animal models, by developing pharmco-therapeutic agents as the background of future clinical trials.[4]

Based on International Society for Hepatic Encephalopathy and Nitrogen Metabolism (ISHEN)14 recommendations, a toxin model of type A hepatic encephalopathy was selected using thioacetamide (TAA). The model is very similar to human acutely progressive hepatic disorders with the parallel involvement of the brain.[14] Thioacetamide causes hepatocellular necrosis, bridging necrosis and lymphocytic infiltrate without any cholestasis. This model has been used to clarify changes in the functions of the CNS in HE.[7][15] This study determines the behavioral, biochemical and histological aspects of acute hepatic encephalopathy in rat as an animal model of the disease.

## Materials and Methods

We designed a systematic animal study to find out which laboratory rodent and gender and what doses of TAA were practically more appropriate to induce acute hepatic encephalopathy (AHE). In phase 1; 3 available species of rodents (C57BL6, BALB/C mice and Sprague Dawley rats; 10 animals in each group) were used for induction of AHE to clarify which one was the best animal of choice. The dosage of TAA was selected according to the literature (300 mg/kg/day at 24 h intervals for 4 days).[12] The animals received intraperitoneal injections of thioacetamide. The animals were kept at 12 hours light and 12 hours darkness, temperature of 22°C, humidity of 30%. All animals had free access to food and water. All experimental animal protocols were approved by the Ethics Committee of Shiraz University of Medical Sciences. Animal selection, all experiments, subsequent care and the sacrifice procedure were all adhered to identical guidelines under supervision of Animal Care Committee of Iran Veterinary Organization. All experiments were carried out under aseptic conditions in Laboratory Animal Center of Shiraz University of Medical Sciences. The induction rate and the mortality rate were recorded. The lowest morality rate was observed in Sprague Dawley rats after administration of TAA, so this species was selected for the next step of the experiment.

In phase 2; to compare the effect of gender of rats in the modeling of AHE by TAA, both male and female genders of Sprague Dawley rats, weighing 180-200 g were enrolled. The groups were compared for mortality rate and the clinical grading by the scoring method represented in Table 1.

**Table 1:** Clinical grading scores of the animals’ behavior

**Clinical grade**	**Definition**
0	Normal behavior
1	Mild lethargy
2	Decreased motor activity, poor gesture control, diminished pain perception
3	Sever ataxia, no spontaneous righting reflex
4	No righting reflex, no reaction to pain stimuli

In phase 3; after selection of male gender as appropriate one, different doses of the TAA were compared in male Sprague Dawley rats. The animals (10 male rats in each group weighing 180-200 g) were categorized in 4 groups according to the dose of the TAA administered. Each group received intraperitoneal injections of one of the three doses (200, 300 and 400 mg/kg) of TAA at 24 h intervals for 4 days. A control group was treated with solvent of TAA which was water (5 ml/kg/day) (Table 2). The animals also received dextrose water and ringer lactate solutions (10 mg/kg/day, IP) to prevent renal failure, hypoglycemia and electrolyte imbalance till the end of the experiment.

**Table 2:** Groups receiving TAA or solvent in phase 3.

**Group**	**TAA Dose (mg/kg)**	**No.**	**Weight (Mean, g) **	**Gender**
1	0	5	186	Male
2	200	10	191.5	Male
3	300	10	189.2	Male
4	400	10	184.7	Male

The animals were weighted and clinically evaluated in a daily manner. The onset of behavioral (clinical) signs of encephalopathy in different TAA dosage groups were compared. In this phase, the mortality rate, the daily animal weight loss and the clinical grade were evaluated. On day 4, the blood samples of animal who survived the experiment were sent to laboratory for evaluation of biochemical markers of hepatic failure including alkaline phosohatase (ALP), alanine transaminase (ALT) and total bilirubin. Samples were also sent for determination of blood ammonia level (NH4L) urgently. The livers were removed and transferred into 10% formalin for histological evaluations.

In phase 4; 180-200 g male Sprague Dowley rats (N=45) were enrolled to induce AHE and were evaluated for clinical grade of AHE and the correlation between this score and the biochemical markers and histological findings. So a dose of 300 mg/kg/day of TAA were administered for 4 days. On the 4th day, the rats were divided into four groups in accordance with the attributed clinical grade. Blood samples were provided and the tests were repeated as mentioned in step 3. After scarification of the rats, livers were removed and immediately placed in 10% neutral buffered formalin. Tissues were then embedded in paraffin and 5 µm thick sections were prepared, dehydrated and stained with hematoxylin and eosin and Masson trichrome dyes. Two pathologists who were blind to the groups reported the results based on Bruck scoring method.[16]

SPSS software (Version 15, Chicago, IL, USA) was used for statistical analysis. Mortality was expressed as a percentage of the total group number for each treatment regime. ANOVA and Scheffe as post hoc analysis were used to determine any differences between groups after testing normality for each biomarker. In the case that ANOVA showed statistical significance, the LSD procedure was used for individual group comparison. Levels of significance were set at P<0.05. To examine the correlation between each biomarker and the clinical grade, Pearson correlation test was implemented.

## Results

In phase 1, the best animal species were enrolled which were able to tolerate the induction of AHE by TAA (300 mg/kg). The induction rate and the mortality rate in each group were presented in Table 3.

**Table 3:** The induction and the mortality rates in 3 species of rodents.

**Species**	**Induction rate (%)**	**Mortality rate (%)**
Rats	80	10
C57/BL6 mice	70	40
BALB/C mice	80	50

Due to lower mortality rate, Sprague Dawley rats were selected for the next step. In phase 2; both genders showed signs of encephalopathy and the rate of successful induction as well as mortality rate were similar. The mean clinical scores did not show any significant difference between male and female rats.

In phase 3; for dose of 400 mg/kg; 70% of rats developed clinical score of 1 within 18 hours after the 1st injection. In the two other TAA groups till 18 hours, the percent of induced rats presenting grade 1 were 50% and 30% for 300 and 200 mg/kg respectively. In the 3rd day post-induction, all three groups of TAA showed various higher grades of encephalopathy.

The mortality rate in the group receiving 400 mg/kg of TAA was unexpectedly high (40%). The mortality rate was 10% in both groups receiving 200 and 300 mg/kg of TAA. In all groups, the laboratory data was taken only from survivors. The mean clinical grade, biochemical markers for hepatic dysfunction, blood ammonium level and pathological scoring were represented in Table 4.

**Table 4:** The mean clinical grade, biochemical markers for hepatic dysfunction and blood ammonium level of survivor rats in each study group.

**Dose of TAA**	**Clinical score**	**NH3 level**	**AlP**	**SGOT**	**SGPT**
0	0	98.6	31	63	46.8
200	0.89	1176.56	381	1328	433.33
300	2.4	917.11	712.78	1249	858.56
400	2.83	1183.5	896.33	1270	957.17

The control group (not receiving TAA) was significantly different with the induced groups (receiving different doses of TAA) regarding clinical scores, all biomarkers and histological findings. There was difference between clinical scores in induction groups (induced by different dosages of TAA). Clinical scores of AHE group induced with 200 mg/kg of TAA and higher doses (300 and 400 mg/kg) were significantly different (p=0.029 and 0.001 respectively), but there was no difference between the clinical scores in groups receiving 300 and 400 mg/kg of TAA. As the mortality rate was lower in dose of 300 mg/kg of TAA group with better presentations of the signs and symptoms of AHE; this dosage was considered as the best dose for induction of AHE.

Among TAA receiving groups, no difference was noticed in the level of SGOT and NH3. Although SGPT was not significantly different between 300 and 400 mg/kg of TAA groups, there was a statistically significant lower level of SGPT in 200 mg/kg dose of TAA when compared with groups receiving higher doses. ALP raised with increase of the TAA dose.

In phase 4; the AHE rats receiving 300 mg/kg of TAA were classified according to the attributed clinical score defined in Table 1 (0-4). The frequency of scores in this phase was represented in Table 5.

**Table 5:** The percent of scores in rats induced by TAA (300 mg/kg/day).

**Groups **	**Clinical score**	**Percent of score (%)**
1 (Non-induced)	0	9.1
AHE 2	1	15.9
AHE 3	2	34.1
AHE 4	3	29.5
AHE 5	4	11.4

The biochemical markers and histological findings were compared between each arbitrary group. There was difference between group 1 (grade 0, non induced group) and all other groups (AHE groups 2-5) in their average biomarkers (SGOT, SGPT, ALP and NH3). There was a difference between all groups (p=0.001 for SGOT and 0.004 for SGPT). Among the AHE groups, the only significant difference existed between AHE arbitrary groups 2 and 5, with clinical scores of 1 and 4 respectively (p=0.016 for SGOT and 0.026 for SGPT) (Table 6).

**Table 6:** Correlation between each biomarker and the clinical grade in phase 4.

**Biomarker**	**Correlation coefficient**
SGOT	0.64
SGPT	0.5
ALP	0.38
NH3 Level	0.83

There was a difference between groups for alkaline phosphatase (p=0.002). The difference was seen between non-induced group and AHE groups. Among the AHE groups, there were no significant differences. Regarding ammonium (NH3), there was a difference between groups (p=0.001). Among the AHE groups, the only significant difference existed between AHE groups 2 and 5, with clinical scores 1 and 4 respectively (p=0.01). There were correlation between all biomarkers and the grade of the disease too.

**Fig. 1:**
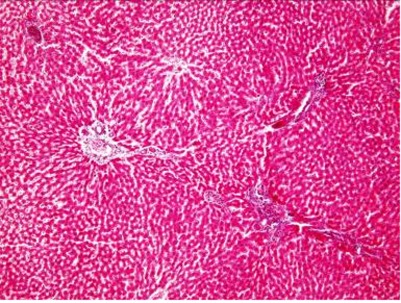
Control group receiving distilled water (x200, H and E).

**Fig. 2:**
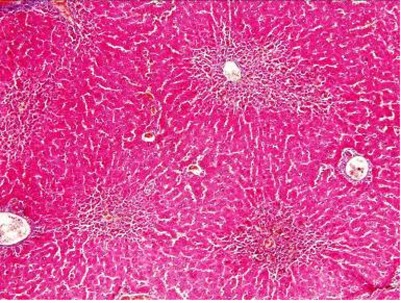
Liver inflammation after administration of 200 mg/kg of TAA (x200, H and E).

**Fig. 3:**
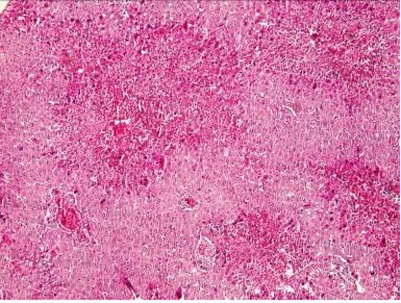
Extensive inflammation, necrosis and fibrosis in liver after administration of 300 mg/kg of TAA (x200, H and E).

**Fig. 4:**
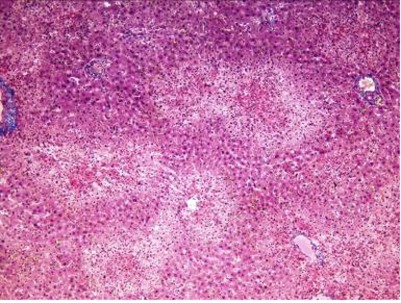
Extensive inflammation, necrosis and fibrosis in liver after administration of 300 mg/kg of TAA (x200, Masson Trichrome).

**Fig. 5:**
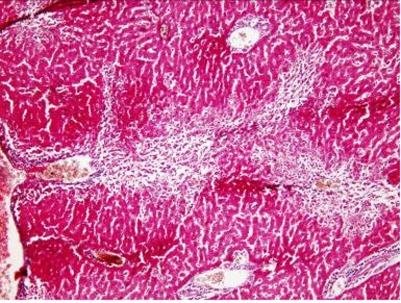
Inflammation, necrosis and fibrosis in liver after administration of 400 mg/kg of TAA (x400, H and E).

Histological findings showed inflammation and necrosis in liver after administration of TAA in centrilobular form after 24 hours and extensive form after 48 hours (Figure 1, Figure 2, Figure 3, Figure 4, Figure 5). As Table 7 shows, the dose of 300 mg/kg of TAA was the best one to induce inflammation, necrosis and fibrosis in liver (AHE) of animals.

**Table 7:** Histological findings after administration of different doses of TAA based on Bruck scoring method.

**TAA Dose (mg/kg)**	**Inflammation (0-3)**	**Regional necrosis (0-3)**	**Extensive necrosis (0-3)**	**Fibrosis (0-3)**
200	1	1	1	0
300	3	3	3	1
400	2	2	2	1

## Discussion

AHE is an emergency condition needing ICU care and is ended in death without liver transplantation.[2] Designing clinical trials on AHE is not easy due to non-homogenous patients under study and little knowledge about the disease pathogenesis and non predictable clinical course.[2] Due to true practical need regarding studying the pathophysiology and pharmacology of acute hepatic encephalopathy, this systematic research on animal models was designed in the animal laboratory of Shiraz University of Medical Sciences. Nemazee Hospital in Shiraz, Southern Iran affiliated to the university is the most important center for liver transplantation in the Middle East[2][3] and has increased the survival of patients suffering from fulminant hepatic failure (FHF) to give them the opportunity of transplantation in a clinical priority.

An animal model can be used for investigation of human diseases for a better understanding of the etiology, pathogenesis, pathophysiology and pharmacology of the diseases without any risk of harm on actual human being during the process of research.[2] Although not perfect,[2] the use of animal models helps the researchers to investigate disease status which might be inaccessible in diseased human.[2] Nevertheless, performing procedures on the laboratory animal imply harm to animal which is not ethical to implement on human.[3][4]

Our laboratory needed its own set up of the model to provide the researchers with some practical characterization of the animal counterpart and getting more valid results in their researches on AHE. The established model could be a basis for future studies on the disease.[17] From the ethical point of view, optimization of the animal models can help in prevention of unnecessary animal death and so saving more laboratory animals. Furthermore, some aspects of this study were novel. When considering the literature, there are no studies comparing different animal models. Due to lower mortality rate, Sprague Dawley rat was used to induce AHE which is superior to induction in mouse, either Albino or C57BL6. The monitoring of behavior is also easier in Sprague Dawley rats. The gender did not play any role in the induction rate of AHE in rats. The mortality rate in both sexes was not different. The best dose of the TAA to induce AHE was 300 mg/kg/day for four days without losing a considerable number of animals for mortality. The majority of animals presented the behavioral scores of 2 and 3 (Table 5). It seems that there was no significant difference among the biological markers of hepatic insufficiency in different groups presenting different clinical scores. In other words, no liver marker can predict the prognosis and outcome of encephalopathy in the TAA model of AHE. Although in the rats presenting normal behavior (clinical score 0, group 1), the difference of biomarkers was significant in comparison with induced groups. Perhaps it could be explained that non-induced animals were relatively resistant to liver failure caused by TAA that can be a matter of further research. Also the rise in a few biomarkers in parallel to increase in behavioral score were noticed when the worst clinical condition (score 4) was compared with the minimal behavioral disaster (score 1) even this was not consistently seen for all measured enzymes and markers. This finding was never reported before in TAA induced AHE which is a known fact in human AHE demonstrating that the model was a good biochemical simulation of the human disease condition. There was a positive correlation between behavioral scores clinically and the level of biomarkers in the blood. It might suggest that these biochemical markers can be followed as acceptable biomarkers when the experimental drugs were tried on the model. The future studies on the model can be equipped with behavioral measurement by accepted behavioral systems to enrich the studies focused on encephalopathy. Neurophysiological and neuropathological assessment of the CNS of the induced rats can also be used in neuroscientific studies of AHE. The model can also be used for molecular, biological and immunological studies of the disease. Many neuroprotective as well as hepatoprotective agents can be tried mechanistically as pharmacological tools. These approaches might sometimes fruit and the translational medicine based on the AHE model can practically help to increase their opportunity to survive till the appropriate liver could be donated for transplantation.
